# miPepBase: A Database of Experimentally Verified Peptides Involved in Molecular Mimicry

**DOI:** 10.3389/fmicb.2017.02053

**Published:** 2017-10-23

**Authors:** Anjali Garg, Bandana Kumari, Ravindra Kumar, Manish Kumar

**Affiliations:** Department of Biophysics, University of Delhi, New Delhi, India

**Keywords:** autoimmune disease, molecular mimicry, database, peptide, cross-reactivity

## Abstract

Autoimmune diseases emerge due to several reasons, of which molecular mimicry i.e., similarity between the host's and pathogen's interacting peptides is an important reason. In the present study we have reported a database of only experimentally verified peptide sequences, which exhibit molecular mimicry. The database is named as **miPepBase (Mi**micry **Pep**tide Data**base**) and contains comprehensive information about mimicry proteins and peptides of both host (and model organism) and pathogen. It also provides information about physicochemical properties of protein and mimicry peptides, which might be helpful in predicting the nature of protein and optimization of protein expression. The **miPepBase** can be searched using a keyword or, by autoimmune disease(s) or by a combination of host and pathogen taxonomic group or their name. To facilitate the search of proteins and/or epitope in miPepBase, which is similar to the user's interest, BLAST search tool is also incorporated. **miPepBase** is an open access database and available at http://proteininformatics.org/mkumar/mipepbase.

## Introduction

Mimicry is a very common phenomenon in which a living being pretends to be what it is not. By adopting mimicry, an animal get protection by not hiding, rather being mistaken for something a predator will avoid because either it look dangerous or tastes bad. Hence, it is not surprising that similar strategy has been exploited at the molecular level as well. The obvious benefit molecular mimicry confers to pathogens is to fool the host's defenses and survive. The presence of a molecule in a pathogen that is similar with a host antigen could inhibit the immune response of the host against the pathogen because of the immune tolerance toward self-antigens (Davies, 1997; Gowthaman and Eswarakumar, 2013). For example, *Helicobacter pylori* infection in human triggers two autoimmune diseases namely autoimmune gastritis and pernicious anemia. It occurs because activated CD4^+^ Th1 cells infiltrates into gastric mucosa and they cross-recognize the self-epitopes of H^+^K^+^ ATPase and *H. pylori* antigens (D'Elios et al., 2004).

There are number of well documented molecular mimicry events, using which bacteria, viruses, or parasites evade the host's immune response (Oldstone, 2005). The pathogen's protein having similar epitope to that of the host results in cross-reactivity that generates immunological response against self (i.e., host), which ultimately leads to autoimmune diseases (Oldstone, 1998; Cusick et al., 2012). The peptides, which display this property, are called mimicry peptides and the phenomenon is called molecular mimicry (Davies, 1997). The role of molecular mimicry in autoimmune disease was getting strengthen when it was observed that the antibody against the phosphoprotein of measles virus and Herpes simplex type I can cross-react with human intermediate filament protein vimentin (Fujinami et al., 1983). Molecular mimicry can cause several immune-mediated disease such as Grave's disease (Kohn et al., 2000; Chen et al., 2001), Insulin-dependent diabetes (Rose and Mackay, 2000; Hiemstra et al., 2001), Multiple sclerosis (Banki et al., 1994; Wucherpfennig and Strominger, 1995; Appelmelk et al., 1996; Talbot et al., 1996; Rose and Mackay, 2000), Peptic ulcer (Appelmelk et al., 1996), Rheumatoid arthritis (Tiwana et al., 1999; Balandraud et al., 2004; Bridges, 2004), Systemic lupus erythematosus (Rönnblom and Alm, 2001; Kaufman et al., 2003; McClain et al., 2005), Myocarditis (Neu et al., 1987; Huber et al., 1994; Gauntt et al., 1995; Schulze and Schultheiss, 1995; Ang et al., 2004), and cancer as well, by modulating key signaling pathways, such as those involving Ras (Guven-Maiorov et al., 2016). A number of studies have deciphered various prospects and aspects of molecular mimicry, but these are scattered in numerous research papers. Compilation of the available information from literature can greatly facilitate the researchers who work in this domain. At present there is no data repository, which contains all the information related to autoimmune diseases caused due to molecular mimicry because piecing, together of this scattered data and discerning the accompanying details is complicated and tedious. To the best of our knowledge, only one database namely mimicDB (Ludin et al., 2011) is available which provides information about proteins or epitopes involved in host-pathogen interactions. But mimicDB is restricted to information pertaining to only a few human parasites. Also, the mimicry candidates of mimicDB were predicted through a computational pipeline.

**Figure 1:**
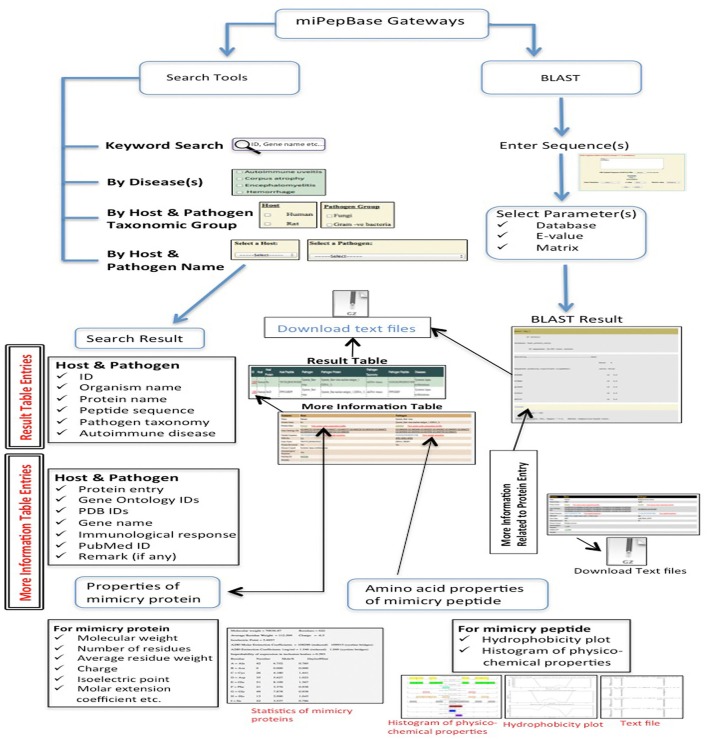
Architecture of miPepBase.

In the present study we have reported a freely accessible database, which can serve as a comprehensive and high quality resource of peptides involved in molecular mimicry. We have also incorporated the information related to autoimmune diseases as well as in-depth information about mimicry peptide and proteins. The database is named, **miPepBase (Mi**micry **Pep**tide Data**base**), which is available at http://proteininformatics.org/mkumar/mipepbase. All molecular mimicry based autoimmunity events compiled in miPepBase were experimentally verified by the respective researchers and are supported by peer-reviewed publications. **MiPepBase** is an open access database that provides comprehensive information about the mimicry proteins and peptides of both host (and model) and pathogen. The information includes the names of host and pathogen proteins, sequences of mimicry peptide, autoimmune disease caused due to mimicry peptide, gene ontology information of the protein, PDB ID of the structure of protein (if present), type of immunological response generated by mimicry peptide and much more. We anticipate that miPepBase will help researchers to generate new hypothesis about different aspects of molecular mimicry and also act as a unified resource of information about molecular mimicry. The miPepBase can be searched using keyword(s) or by autoimmune disease(s) or by a combination of host and pathogen taxonomic groups or their names. The database also includes BLAST search tool to facilitate sequence similarity search against the mimicry proteins and/or peptide contained in it. Each miPepBase entry is also linked to many popular global repositories such as UniProt (Apweiler et al., 2004), PDB (Berman et al., 2000), EMBL-EBI QuickGO (Binns et al., 2009), and PubMed. MiPepBase also provides information about physicochemical properties of proteins containing mimicry peptides, which might be helpful in predicting the nature of protein and optimization of its expression. The basic architecture of miPepBase is shown in Figure 1. The data of miPepBase can also be downloaded in text file. Overall, mimicry peptides which are compiled in miPepBase might help in opening new gateways to explore the role of molecular mimicry in autoimmune diseases that are yet unaddressed. It is anticipated that miPepBase would be helpful in understanding the details of molecular mimicry and expedite the process of disease detection, diagnosis, prognosis, and even deciding the therapeutic regimen of autoimmune diseases.

## Materials and methods

### Data collection and compilation

The main aim of miPepBase was to collect, compile and curate all the information related to autoimmune disease caused by molecular mimicry. Therefore, experimentally verified data was collected after an extensive search of published research papers with the help of PubMed and Google Scholar using keywords “molecular mimicry,” “host-pathogen cross-reactivity,” and “autoimmune diseases.” We also mined other additional relevant information such as gene and protein names, mimicry peptide sequence, name of autoimmune diseases, and immunological response by T-cells or antibodies. The information regarding proteins, taxonomic classification of pathogen, gene ontology information, PDB ID, annotation status of protein (review status) and protein sequences was obtained from the UniProt protein repository. The miPepBase also provides PubMed link with each entry from which the molecular mimicry and autoimmune disease information was extracted.

### Web interface and database architecture

The inner framework of miPepBase is built using MySQL (http://www.mysql.org), Perl (http://www.perl.org), and Apache (http://www.apache.org) on Cent OS Linux platform. The interface component consists of webpages designed in HTML/CSS in a Linux environment. To provide convenience in usage, the database was developed in a user-friendly manner. The “Browse” and “Search” options were provided to search and access the information content of miPepBase. The home page of miPepBase has a very short introduction about molecular mimicry based autoimmune diseases. It also provides a brief description of the database content and clickable icons with direct links to the database and its different utilities.

### Database accessibility

The miPepBase provides interactive access to the data and the users can connect and access the database using any one among different search options. The search options have been designed in a simple and intuitive manner so that the users can search the database either by keyword or predefined combinations of fields (advanced search).

**Keyword search** assists users to search the database by following fields: database ID or organism's name or protein's name or entry or autoimmune disease or UniProt ID or taxonomic classification or gene ontology ID or PDB ID or peptide sequence or PubMed ID. It also permits free-floating Google like search over entire database.

**Advanced search** provides three different types of search options for users to access the data: First, search by one or multiple autoimmune disease(s) caused due to molecular mimicry. Second, search on the basis of host and pathogen taxonomic group, which allows users to explore one or multiple host(s) and pathogen taxonomic group(s) involved in molecular mimicry. The third and last option of advanced search is a drop down menu of host and pathogen name, which allows searching restricted to a specific set of host and pathogen. Irrespective of the mode of search chosen to query the miPepBase, the search result will be displayed in the tabular format. In the search result, the ID (shown in red color) is a clickable link and can display detailed information of corresponding entry. All the information can be downloaded in the text format, using the “download button” in result table. Additionally, different information related to protein sequence, structure, gene ontology and source of article, were linked to UniProt, RCSB PDB, EMBL-EBI QuickGO, and PubMed, respectively. A detailed step-by-step manual is also provided to assist users in smooth and efficient searching of miPepBase.

### Tools integrated in miPepBase

Different tools are also incorporated in the miPepBase to help users to search related proteins and/or peptides and analyze their different physicochemical properties. BLAST searches similar sequence(s) with in the database (Altschul et al., 1997, 2005) while pepstats and pepinfo utilities of EMBOSS package provides information about physicochemical properties of protein and peptides (Rice et al., 2000). The information derived from these tools might be helpful in predicting the nature of protein and optimization of protein expression.

**Pepstats** was used to calculate physicochemical properties of amino acids (such as molecular weight, number of residues) present in mimicry protein.

**Pepinfo** was used to calculate properties of mimicry peptide which include two types of plots: (i) Hydrophobicity plot (on the basis of Kyte and Doolittle parameters) and (ii) Histogram of presence of amino acid with the physico-chemical properties such as tiny, small, aliphatic, aromatic, non-polar, polar, charged, positive, and negative.

**Basic Local Alignment Search Tool (BLAST):** It is incorporated to find homologous sequence(s) and similar peptide(s) present within miPepBase database. User has to simply paste the sequence in the text box or upload sequence in the FASTA file to find similar sequence(s). Option to specify search parameters like database, *E*-value cutoff and alignment scoring matrix value is also present. The default cut-off *E*-value is 100 and alignment-scoring matrix is BLOSUM62. In the miPepBase BLAST tool, four different types of databases namely Host protein, Host peptide, Pathogen protein, and Pathogen peptide are present. Hence, similarity search can be carried out against any of the four databases.

## Results

### Data statistics and content

**Figure 2:**
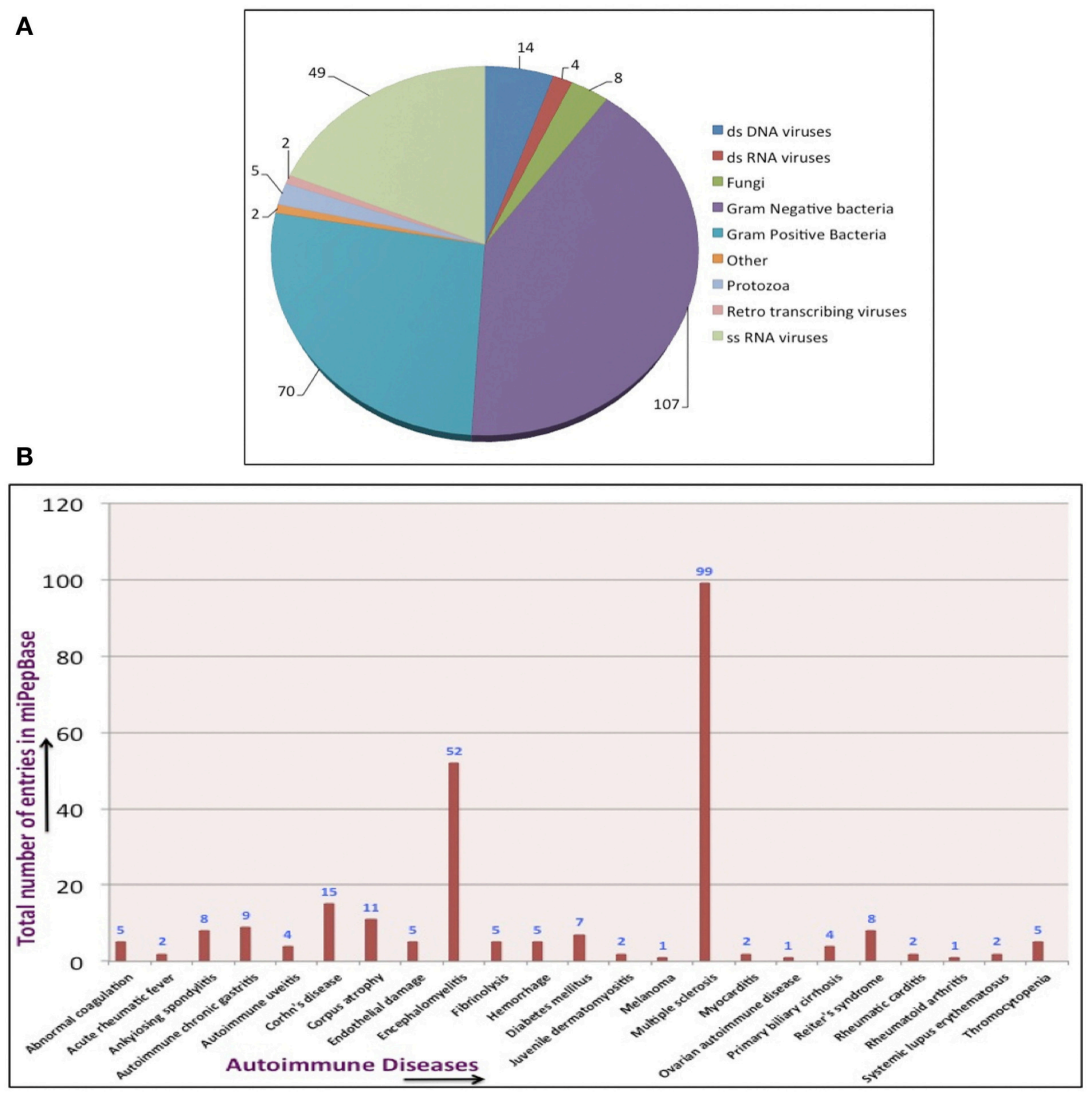
Data statistics **(A)** Based on pathogen taxonomic group, **(B)** Based on autoimmune disease.

In the miPepBase, only experimentally verified mimicry peptides from published papers are incorporated. The first release of miPepBase has 261 entries in total. It does not mean that miPepBase contains 261 host-pathogen peptide pairs. This is due to existence of multiple mimicry peptides in a single protein. Analysis of the miPepBase data shows that in both host and pathogen proteins more than one stretch of amino acids might be involved in molecular mimicry. The following information is associated with each entry:

**ID:** It is a unique identifier assigned to each entry of the miPepBase database. Each ID is linked to the detailed information of that entry, which includes details of host and pathogen proteins, their gene ontology information, PDB ID of structure (if known), gene name, annotation status of protein (reviewed/not reviewed), PubMed ID, and remark (if any).
**Organism's name:** With each event of molecular mimicry two different organisms are associated. Organism in which autoimmune response is generated was designated as host. Organism, which encodes the mimicry peptide, was designated as pathogen.
**Protein names:** Two different proteins are associated with each event of molecular mimicry. One that is encoded by the host and second which is encoded by the pathogen. Names of both the proteins are present with each entry.
**Peptide sequence:** This contains the stretch of amino acids (the peptide) present in both host's and pathogen's protein that actually leads to molecular mimicry.
**Pathogen taxonomic group:** Organisms from all taxonomic groups such as bacteria, viruses, fungi, and protozoa exhibit molecular mimicry. MiPepBase contains information of molecular mimicry based autoimmunity events caused by organisms from all taxonomic groups.
Broadly, pathogens are divided into four taxonomic groups namely bacteria, fungi, protozoa, and viruses. Bacteria is further subcategorized into gram-positive, gram-negative, and others i.e., diderms. Further, viruses are categorized according to the classification system purposed by David Baltimore (reviewed in Baltimore, 1971), namely retro transcribing virus, dsDNA virus, dsRNA virus, and ssRNA virus. The total numbers of entries belonging to pathogens of different categories is shown in Figure 2A.
**Autoimmune disease:** This field provides the information about disease caused due to molecular mimicry. Our analysis revealed that very diverse types of autoimmune diseases might occur due to molecular mimicry. Data content of miPepBase shows total 23 types of autoimmune diseases are associated with molecular mimicry. Multiple sclerosis was the most frequent disease followed by encephalomyelitis. The different types of autoimmune diseases and the number of times they were associated with molecular mimicry is shown in Figure 2B.

### How to search query into miPepBase?

#### Using a keyword

**Figure 3:**
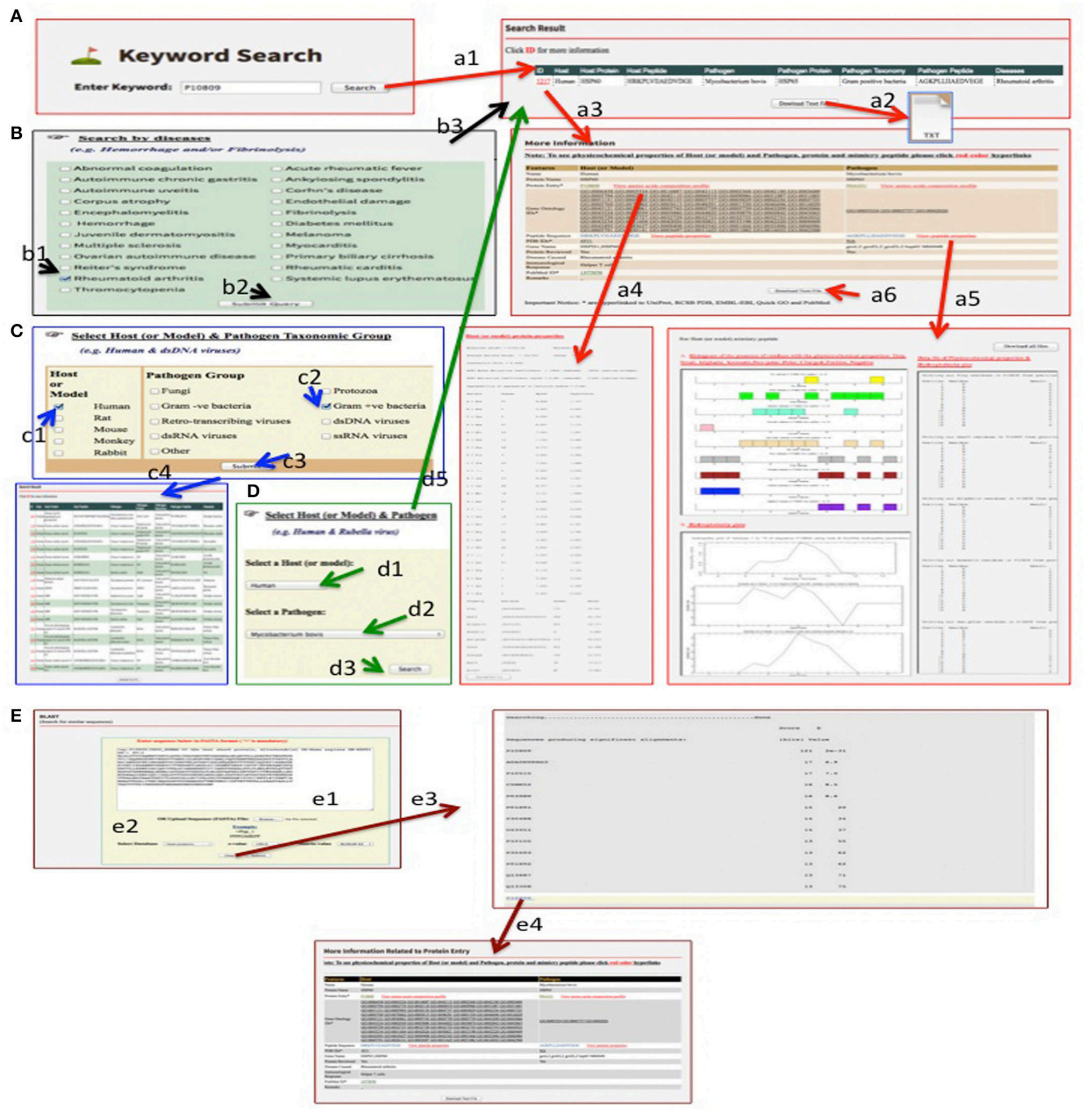
Process of stepwise data retrieval and analysis in miPepBase. The user can search query with following options: **(A)** Keyword search, **(B)** Search by disease, **(C)** Search by host and pathogen taxonomic group, **(D)** Search by host and pathogen name. The search from **(A–D)** options display search result table and from that user can select the entry/displayed result for further detailed analysis. Sequence based search can also be searched by **(E)** BLAST search option and each hit is further linked to its details information page. The detail of result obtained from search options **(A–E)** is displayed by corresponding small case (the number indicates step number). From example a1–a6 denotes the results that can be obtained using keyword search option **(A)**.

Any data in miPepBase can be search and access by five different ways. It is illustrated here using one protein (UniProt accession number **P10809)**. Users can get the information associated to this protein by querying miPepBase submission of UniProt accession number as a keyword to the “Keyword search option” (Figure 3A) and click the search button (Step a1). The search result page showed a single hit and the information related to P10809 protein was presented in tabulated form. The search result contains following information: unique miPepBase ID (1217), host name (human), host protein name (HSP60), host mimicry peptide sequence (HRKPLVIIAEDVDGE), pathogen name (*Mycobacterium bovis)*, pathogen protein name (HSP65), pathogen taxonomy (Gram positive bacteria), pathogen mimic sequence (AGKPLLIIAEDVEGE), and autoimmune disease (Rheumatoid arthritis) caused due to host and pathogen cross reactivity. All these details can also be downloaded as text file (Step a2). More detailed information related to P10809 can be retrieved through miPepBase ID of P10809 (i.e., 1,217, displayed in red font in the search table) (Step a3). Further, it will give more information about the host's and pathogen's: protein entry (host-P10809 and pathogen-P0A521), gene ontology (available for both), PDB ID (host- 4PJ1 and pathogen-NA), gene name (host-HSPDI, HSP60 and pathogen-groL2, groEL2, groEL2, hsp65, Mb0448), protein reviewed (host-yes and pathogen-yes), immunological response (Helper T cell), PubMed ID (1577070), and remark (NA). In addition to these details the miPepBase also provide direct link to UniProt, EMBL-EBI, RCSB PDB, and PubMed. All information described above can also be downloaded as “Text File” (Step a6).

Apart from above described information users can also get the amino acids composition profiles for P10809 (host's protein) and P0A521 (pathogen's protein) entries and their hydrophobicity graph and other physico-chemical information for mimicry peptides through “View amino acids composition profile” (Step a4) and “View peptide properties” (Step a5), respectively. All graphs and text file related to physico-chemical properties of protein and peptide can be downloaded in text format.

#### By disease

To retrieve the information related to mimicry proteins involved in a particular set of autoimmune diseases, users could use an advanced search option i.e., “Search by Diseases.” This option lists a set of disease caused due to molecular mimicry and whose information is present in miPepBase. Here, it is demonstrated using **Rheumatoid arthritis** as an example (Figure 3B). On selection of rheumatoid arthritis as the disease whose information is desired (Step b1 and b2), search result page (Step b3) would be displayed. The search page would list the information related to proteins involved in the rheumatoid arthritis in a tabulated form. The information content and ways to navigate different sections remain same (Step a3–a6) as explained above for P10809 protein using Keyword search option.

#### By host and pathogen taxonomic group

This option provides a list of pathogens and host taxonomic group within which the search will be restricted. This search option gives an easy way to do comparative analysis among mimics encoded by different pathogens of same or different taxonomic group(s) (Figure 3C). Searching (Step c3) with “**Human**” **as host** (Step c1) **and “Gram-positive bacteria” as pathogen taxonomic group** (Step c2), total 19 entries related to gram-positive bacteria group (Step C4). Here also the presentation of search result data and further information (from Step a3 to a6) were remaining same as discussed for above two searching methods.

#### By host and pathogen name

The information related to event of cross reactivity between a specific host and pathogen that leads to autoimmune disease(s) can be achieved using another advanced options i.e., “Select host and pathogen.” The names of host and pathogen can be selected from the dropdown menu present in this section. Here, it is exemplified (Figure 3D) using **Human as host** (Step d1) **and** ***Mycobacterium bovis* as pathogen** (Step d2). After submission of query (Step d3) a result page would be display that contains the search result information in tabulated form. The information content of search page will remain same as explained earlier for keyword search (Step a3–a6).

#### By blast search

This is not a direct way to search the data content of miPepBase. Rather it searches similar sequences and peptides in the miPepBase. The BLAST search option is available at menu bar (Figure 3E). The query sequence in FASTA format can either be pasted in the text box or uploaded as sequence file (Step e1). Three parameters have to be optimized for efficient BLAST search (i) the database in which related sequence will be searched; (ii) *E*-value, and (iii) Scoring Matrix. The default *e*-value and scoring matrix are 100 and BLOSUM 62, respectively (Step e2). As shown in section E, when **P10809** protein sequence was searched against host protein database, total 13 hits were obtained which are arranged on the basis of ascending *e*-value (Step e3). Also every BLAST hit protein entry is further linked (in blue color) to detailed information page, which provide tabulated detailed information of corresponding BLAST hit (Step e4). These information are the same as described above for keyword search option (Step a4–a6).

## Discussion

During the last few years, much active research and experimental verification has shed light on various aspects of molecular mimicry and it's role in autoimmune diseases. With the passage of time, number of autoimmune diseases caused due to molecular mimicry is increasing. Since, a unified repository of the available information related to molecular mimicry based autoimmune diseases is not available, hence we have built a database (miPepBase) which not only contains the information regarding proteins and peptides associated with the process, but several other important details also. In-depth analysis of this information might lead to the elucidation of mechanisms of autoimmune diseases controlled by mimicry peptides. Each entry in the miPepBase database is linked to many other molecular biology data repositories. Further, the database also includes inbuilt tools, which can help to fetch other relevant information related to the mimicry proteins and peptides. As more data will accumulate by the use of high throughput molecular, genomic and metagenomic methods, we anticipate that the release of miPepBase will facilitate comprehensive analyses of different factors involved in autoimmune diseases caused by the mimicry peptides. We also hope that miPepBase would be helpful for the scientific community in understanding the host-pathogen interactions, as well as how the pathogens evade host immune systems.

## Comparison with other available database of antigenic peptides

Several web-based antigen/epitope databases are available the content of which is freely available to the users. A brief description of **MimicDB** along with comparison with miPepBase is as follows:

### MimicDB

mimicDB (Ludin et al., 2011) is a database of linear amino acid epitopes derived from a comparative genomics approach. These epitopes were predicted to be a potential molecular mimicry peptide and derived from a computational prediction pipeline. Further mimicDB is focused on a few selected human endoparasites namely *Brugia malayi, Schistosoma mansoni, Plasmodium falciparum, Leshmania major, Cryptosporidium parvum, Trichomonas vaginalis*, and *Trypanosoma cruzi*. In miPepBase the information is not restricted to any particular class of pathogen and/or disease. It contains information related to all autoimmune diseases caused by pathogens, which may belong to viruses, or prokaryotes, or eukaryotes. miPepBase host's and pathogen's mimicry peptides were curated from literature. The respective researchers have already experimentally established the role of these mimicry epitopes in generating autoimmune disease.

## Limitations and future prospects

Although, we have made outmost effort to compile all available data at one place, it cannot be claimed that miPepBase contains information about each and every peptide/protein involved in molecular mimicry based autoimmune diseases. It is certainly possible that few peptides might have been missed and not included in the miPepBase. In future, we would make our best efforts to include the missing as well as newly added data in miPepBase. The motivation behind establishment of miPepBase was to establish a knowledgebase for proteins/peptides involved in molecular mimicry. We will continue to add new information, which may include but not limited to interaction partners of mimicry proteins and their role in disease. This will enable us to provide a platform for study of the mimicry peptides and pathways through which they trigger autoimmune diseases. We believe the miPepBase database would helpful to the scientific community in exploring the various prospect and aspects of molecular mimicry.

## Database update

An important aspect of any database is to keep it up to date by adding new data. We would constantly add information about newly discovered peptides, which exhibit molecular mimicry and cause autoimmune diseases.

## Accessibility and data download

The database and its contents are freely accessible without any restriction at http://proteininformatics.org/mkumar/mipepbase.

## Author contributions

AG and BK prepared the manuscript, collected and organized the data. AG and RK developed the web interface. AG, BK, RK, and MK analyzed the data. MK conceived the idea. All authors reviewed the manuscript.

### Conflict of interest statement

The authors declare that the research was conducted in the absence of any commercial or financial relationships that could be construed as a potential conflict of interest.
